# Local Area Water Removal Analysis of a Proton Exchange Membrane Fuel Cell under Gas Purge Conditions

**DOI:** 10.3390/s120100768

**Published:** 2012-01-11

**Authors:** Chi-Yuan Lee, Yu-Ming Lee, Shuo-Jen Lee

**Affiliations:** Department of Mechanical Engineering, Yuan Ze Fuel Cell Center, Yuan Ze University, Taoyuan 320, Taiwan; E-Mails: s939202@mail.yzu.edu.tw (Y.-M.L.); mesjl@saturn.yzu.edu.tw (S.-J.L.)

**Keywords:** gas purge, fuel cell, high frequency resistance, micro sensor

## Abstract

In this study, local area water content distribution under various gas purging conditions are experimentally analyzed for the first time. The local high frequency resistance (HFR) is measured using novel micro sensors. The results reveal that the liquid water removal rate in a membrane electrode assembly (MEA) is non-uniform. In the under-the-channel area, the removal of liquid water is governed by both convective and diffusive flux of the through-plane drying. Thus, almost all of the liquid water is removed within 30 s of purging with gas. However, liquid water that is stored in the under-the-rib area is not easy to remove during 1 min of gas purging. Therefore, the re-hydration of the membrane by internal diffusive flux is faster than that in the under-the-channel area. Consequently, local fuel starvation and membrane degradation can degrade the performance of a fuel cell that is started from cold.

## 1. Introduction

Water content is a key parameter in a fuel cell that is operated under low temperature. The proper water content in a fuel cell will reduce the resistance of the membrane and promote the electrochemical reaction in the cell. However, when the rate of water production in a fuel cell exceeds its removal rate, liquid water accumulates in the membrane, catalyst layer, gas diffusion layer (GDL), and even flow channel. Flooding with water influences the performance of the fuel cell, causing corrosion of the carbon in the catalyst layer owing to fuel starvation in the porous structures that are blocked by the water. Furthermore, at sub-freezing temperatures, ice/frost is formed from the residual liquid water, blocking the pores of the membrane electrode assembly (MEA), seriously affecting the ability of the fuel cell to start from cold. Various studies have focused on degradation of cell material by flooding with water and fuel starvation [1,2].

Gas purging is an effective procedure for removing residual fuel and liquid water from a cell, and has been adopted as a crucial procedure in a PEM fuel cell vehicle (FCV), and is normally repeated in many start and stop cycles. The purpose of gas purging is to remove residual liquid water from all of the compartments in the cell, particularly in preparation of starting from cold at freezing temperatures.

Ge and Wang [3,4] characterized the formation of water/ice in the catalyst layer upon startup of a proton exchange membrane (PEM) fuel cell at subzero temperatures. They found that gas purging was crucial to self-startup at subzero temperatures. During a short period of purging with dry gas, there was substantial relaxation of membrane high frequency resistance (HFR) over a period of more than 30 min, indicating the distribution of water in the MEA. Tajiri [5] proposed an experimental procedure for determining the water content in the fuel cell before and after gas purging. They used the HFR of the cell as an indicator of membrane water content and therefore of the effectiveness of gas purging. They also found that gas purging performance could be uniquely evaluated from the diffusive flux of water vapor across the catalyst layer and GDL, and the convective flux of water vapor along the channel that was purged with gas. The experimental results demonstrate that helium gas purging was superior to nitrogen gas purging as it had three times the diffusivity in water.

A fundamental understanding of gas purging and water removal is necessary. The total water content and the effects of gas purging on the ability of cell to start from cold have been analyzed both theoretically and experimentally [3–10]. However, water content in a fuel cell is strongly affected by the generation of a local current and channel/rib geometry. Non-uniform water distributions may greatly influence the ability of a cell to be started from cold, especially when the non-uniformity is on the scale of a channel or rib area. With respect to the effects of gas purging on a local area, few investigations have focused on the mechanism of local purging and those have involved only theoretical prediction because of the complexities of local measurement methods. Without experimental study, however, the effects of gas purging on a local area remain doubtful.

To diagnose local area cell performance, in most studies [11–13], segmented cells are utilized. Performance is assessed in terms of voltage distribution, current density and membrane resistance from gas inlet to outlet. However, these assessments are made on a large scale, and determining local cell performance under the channels and ribs is difficult. Fundamental transport phenomena still warrant an in-depth investigation.

This study develops a reliable experimental protocol for determining the overall and local water contents following a short-term gas purge. The HFR value of the membrane is an indicator of membrane water content. Gas flow rate and purge duration are key parameters in purging with gas. In this investigation, novel dedicated sensors are utilized for the first time to measure local HFR variations in both along-the-channel and across-the-channel directions under gas purging. The water content is evaluated at various locations in a cell, providing important experimental results. This approach is very useful for fundamental research into the removal of water in a PEMFC by purging with gas.

## 2. Gas Purge

**Figure 1.**
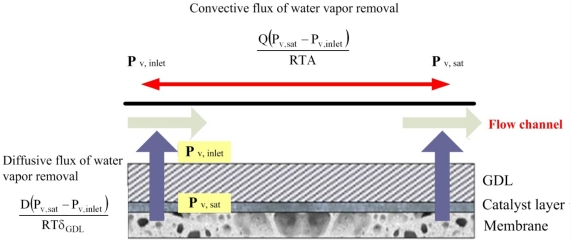
Schematic diagram of water removal during gas purge [5].

The removal of water in an MEA during a purge using gas is assumed to proceed mostly via a vapor phase transport process. Liquid water in a flow channel is immediately removed upon purging with gas. Removing liquid water in the MEA takes much longer as it involves the vapor transport process. Therefore, water is removed from liquid surfaces in MEA to the gas channel by through-plane vapor diffusion and convection. Figure 1 schematically depicts purging with gas.

Tajiri and Wang [5] mathematically modeled water removal by diffusion and convection: through-plane water removal can be characterized by the diffusive flux from a liquid surface in a catalyst layer/GDL to the gas channel:

(1) $$\frac{D\left(P_{v,\text{sat}}-P_{v,\text{inlet}}\right)}{\text{RT}\delta_{\text{GDL}}}$$

where D is the diffusion coefficient of water upon purging with gas; P_v,sat_ is the saturation vapor pressure at the purging temperature; P_v,inlet_ is the partial pressure of the water vapor in the purging gas at the inlet; R is the universal gas constant; T denotes the cell temperature and δ_GDL_ is the effective diffusion path of water vapor from the membrane to the gas channel.

The convective flux of water vapor from the GDL to the flow channel is given by:

(2) $$\frac{Q\left(P_{v,\text{sat}}-P_{v,\text{inlet}}\right)}{\text{RTA}}\;$$

where Q represents the volumetric flow rate of the purging gas and A is the reactive area of the cell. This equation assumes that the exiting purging gas is fully saturated with vapor that has been removed.

## 3. Novel Micro Sensors Development

**Figure 2.**
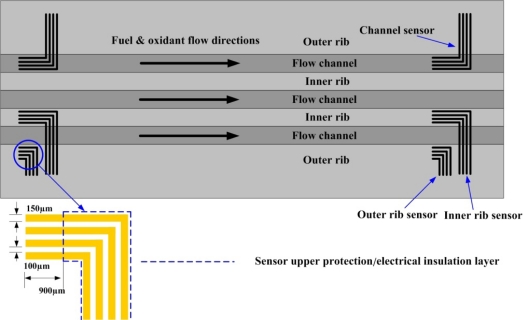
Design of dedicated novel sensors.

**Figure 3.**
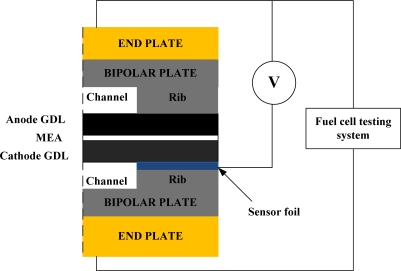
Cross-section of the test single cell.

To diagnose the local membrane high frequency resistance (HFR) variations in both along-the-channel and across-the-channel directions of a PEM fuel cell, micro sensors are designed and positioned along the oxidant inlet and outlet of a straight flow channel. Figure 2 displays the design of the sensor array. The micro sensor measurement area is 900 μm (length) by 150 μm (width). The distance between pairs of adjacent sensors is 100 μm. The substrate of each micro sensor is SS304 stainless steel foil that is 40 μm-thick. The thin micro sensor foil is sandwiched between the cathode GDL and the bipolar plate, as shown in Figure 3.

### 3.1. Micro Sensor Fabrication

**Figure 4.**
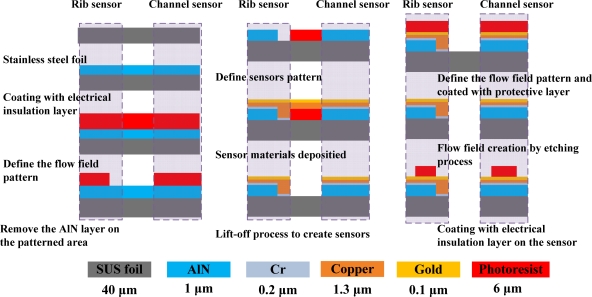
Fabrication of dedicated micro sensors.

**Figure 5.**
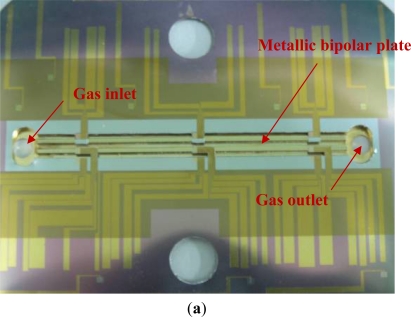

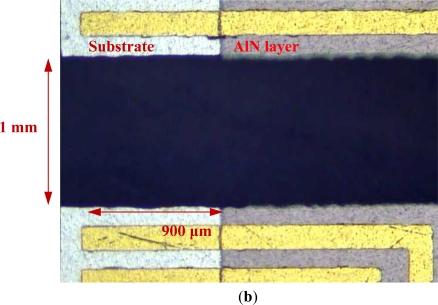
Completed micro sensor foil: (**a**) sensor foil with stainless steel bipolar plate, and (**b**) micro sensors at the under-the-rib area.

**Figure 6.**
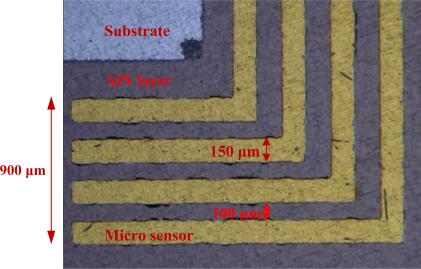
Micro sensors at the under-the-channel area.

**Figure 7.**
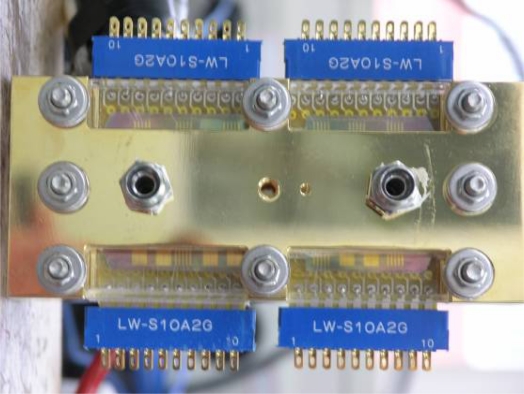
Assembly of single PEM fuel cell with micro sensor foil.

Micro-electro-mechanical systems (MEMS) technology is employed to fabricate micro sensors on flexible SS 304 stainless steel foil. Figure 4 presents the overall fabrication procedure. RCA cleaning was used to eliminate organic matter, oxide layers and ionic contaminants from the stainless steel foil. The aluminum nitride (AlN) electrical insulation coating layer was deposited by RF sputtering. The flow field area of the bipolar plate was patterned on the stainless steel foil using a photolithographic process. The electrical insulation layer in the flow field area was removed by wet etching. Then, photolithography was utilized to define the pattern of the micro sensors and the sensor materials—Cr, Cu and Au—were then deposited by electron beam (E-beam) evaporation. The lift-off process was applied to remove the unwanted sensor material from the stainless steel foil. Finally, the defined flow field area of the bipolar plate on the stainless steel foil was removed by wet etching. Figures 5 and 6 display the micro sensor foil following the MEMS process. The difference between rib sensor and channel sensor is the electrical insulation layer at the rib area was removed due to electrons were transferred by the rib area when the cell was in operation. Figure 7 shows the test single cell. Wire bonding was used to connect the micro sensor to the external circuit.

### 3.2. Experimental Procedure

The reactive area of the cell is 7.2 cm^2^. Both anode and cathode flow fields comprise straight and parallel channels. The bipolar plate is made of SS304 stainless steel with a thickness of 2 mm. The width of each rib and channel is 1 mm. The depth and the length of each channel are 1 mm and 60 mm, respectively. One pair of 30 mm-thick gold-plated copper end plates is placed outside the bipolar plates. The membrane that is adopted herein is commercially available Nafion 212 with a thickness of 50 μm; Pt loading on each electrode is 0.4 mg/cm^2^. The gas diffusion layer (GDL) is Toray carbon paper that is coated with a microporous layer.

The Scribner Associates 850C Compact Fuel Cell Test System is employed to operate the test cell and to control its operating conditions. Variations in the high frequency resistance (HFR) of the internal cell are measured using a functional AC milliohm meter, made by Tsuruga (model 3566) under various gas purging conditions. This meter provides 7.4 mA of AC current with a high frequency of 1 kHz.

Short-term gas purging is employed to remove residual water in the MEA. Therefore, the initial water content in the cell must be well controlled, and the water content is extremely sensitive to the HFR variations. To perform consistent and reproducible experiments to determine the variation in water content with gas purge conditions, a controllable pre-purge condition must be established. The experimental procedure consists of five steps, which are listed in Table 1.

First, the cell is discharged at 0.5 A/cm^2^ for 60 min under fully humidified conditions at 30 °C to ensure that the membrane is fully hydrated. Then, equilibrium purging is carried out to remove all of the liquid water from all of the components of the cell. The operating conditions are nitrogen as the purging gas at a rate of 1 L/min at both the anode and the cathode; purge duration of two hours; a fixed cell temperature of 35 °C; a dew point of the purging gas of 30 °C, and relative humidity maintained at 75%. At the end of this procedure, all of the liquid water in the cell should have been removed and the membrane is in an equilibrium state of 75% RH. At this time, all of the history of the cell is assumed to have been removed, to provide identical conditions across all gas purging experiments. The following step is to set the pre-purge conditions, by generating some liquid water in the cell before it is shut down. The cell is operated at 0.5 A/cm^2^ for 10 min. The cell temperature is fixed at 60 °C and fully humidified. The flow rate of the fuel and the oxidant are fixed at 500 SCCM.

In this stage, all of the pre-purge conditions of the cell apply. Therefore, initial water contents in the MEAs are identical. Although the amount of liquid water in the cell is not easily estimated throughout the pre-purge operation, this procedure provides consistent initial water contents in the MEAs in the subsequent purging experiments. The gas purging experiments are carried out after the pre-purge procedure. One of the key experimental parameters is the purge gas flow rate, which is specified in Table 2. After the gas purging, all of the gas valves are closed and the HFR variations are monitored for 20 min.

## 4. Results and Discussion

### 4.1. Overall Cell HFR Variations

**Figure 8.**
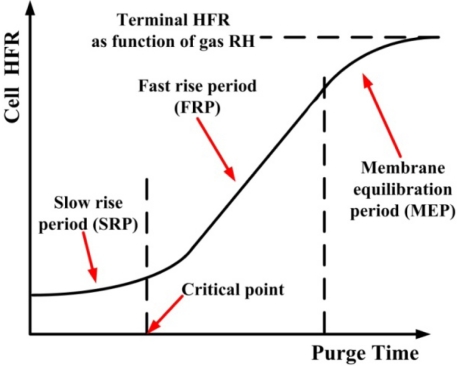
Stages of gas purging [5].

The mechanism of gas purging was proposed by Sinha and Wang [14], and then summarized by Tajiri and Wang [5]. Figure 8 plots a typical gas purging curve. A short-term gas purge can be divided into three major periods.

At the beginning of the gas purge, the GDL and the catalyst layer are dried by the purging gas but the membrane remains hydrated. Thus, the value of HFR remains stable in and this stage is called the slow rise period (SRP). Two drying phenomena occur during the SRP. Through-plane drying occurs first. Liquid water that is stored in the GDL and the catalyst layer is removed or evaporated by the purging gas underneath the channel. Then, water is removed along the in-plane direction from the channel area toward the rib area. At the end of the SRP, all of the liquid water should have been removed from the catalyst layer and the GDL. However, these layers remain saturated with water vapor, and so the membrane remains hydrated. Although the value of HFR remains stable during the SRP stage, it is essential to the removal of all of the liquid water from the MEA. After the SRP stage, the cell HFR increases rapidly. This period is called the fast rise period (FRP). In this stage, more water in the catalyst layer and the membrane is removed by the diffusion of water vapor, until the water reaches equilibrium with the purging gas. After the FRP, liquid water is removed from all of the MEA, resulting in a thermodynamic equilibrium of the water content and the relative humidity of the purging gas is reached; this period is called the membrane equilibrium period (MEP). Hence, the cell HFR is stable again.

**Figure 9.**
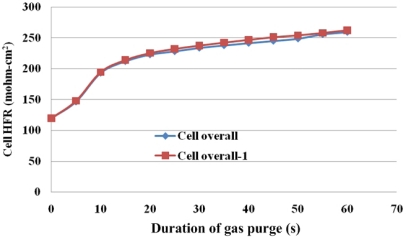
Short-term gas purging with the same purge conditions of 4 L/min.

Figure 9 plots the overall cell HFR variations upon gas purging. The cell temperature is held at 60 °C. The temperature of the purging gas is 41 °C and the flow rate is 4 L/min at both the anode and the cathode. Figure 9 plots repeatably measured gas purging curves, which confirm that the presented pre-purge procedure provides stable initial water content in the MEA. Accordingly, the experiments on the fundamentals of the removal of water by short-term gas purging are reliable.

**Figure 10.**
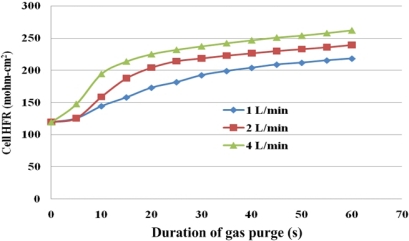
Overall variation of HFR in cell with flow rate of purging rate.

Figure 10 plots the gas purge curve and Table 3 presents the HFR, and the diffusive and convective flux under various gas purging conditions. The final HFR increases with the flow rate of the purging gas. Additionally, an obvious SRP stage is observed when the flow rate was lower than 2 L/min, as shown in Figure 10. As the gas flow rate increases to 4 L/min, the slope of the SRP is greatly increased. These results may demonstrate that as the gas flow rate is increased, the purging gas in the under-the-channel area is more rapidly replenished. Therefore, the water content in the purging gas remains stable and liquid water is continuously removed from the MEA.

As shown in Table 3, convective transport increases the water removal rate. Although the flow rate of the purging gas determines only the convective flux according to Equation (2), at a sufficiently high flow rate, the diffusive transport may also increase with flow rate owing to the increase in the SRP and FRP, as displayed in Figure 10.

### 4.2. Local HFR Variations in Cell

**Figure 11.**
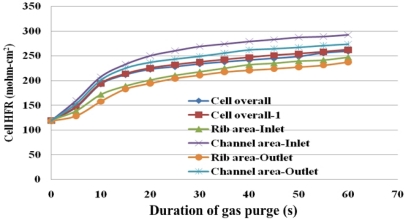
Local HFR variations at flow rate of 4 L/min.

Figure 11 plots the HFR distribution in the cell at a flow rate of the purging gas of 4 L/min. During tests, local HFRs are measured only in the outer rib area and in the channel area. Moreover, only one sensor in the center of each area measures HFR at a particular time because only one local HFR is measured by the high frequency resistance (HFR) meter.

In the across-the-channel direction, the highest final HFR is measured in the under-the-channel area and close to the gas inlet. The lowest final HFR is measured in the under-the-rib area and close to the gas outlet. The purge curve shows a more flat SRP stage takes place at the under-the-rib area. The slope of FRP is lower than that of at the under-the-channel area. These results indicate the membrane drying occurs at the under-the-channel area initially due to difficulty in water removal at the under-the-rib area. Accordingly, the gas purge curve shows a period of rapidly rising HFR in the under-the-channel area. Liquid water stored in the under-the-channel area is immediately removed. Not only water that is stored between the GDL and the catalyst layer but also that in the membrane is removed.

When all of the water that is stored in the under-the-channel area is removed, the membrane drying moves towards the in-plane direction. Figure 11 clearly displays in-plane drying in the under-the-rib area when gas purging has proceeded for more than 5 s. Although the in-plane drying stage follows the through-plane drying, this order is typical of gas purging. However, this is the first time to discover gas purge phenomena under various local areas through the presented experimental procedures and facilities.

Membrane drying in the along-the-channel direction is related to the duration of the gas purging process. In the under-the-channel area, the purge curve shows the value of HFR increases in the along-the-channel direction due to a significant water removal in the gas channel especially for that water stored near the gas inlet section which results in a sharp FRP stage. After more than 20 s of gas purging, the slope of HFR in the FRP decreases and is close to that of the overall cell HFR, as plotted in Figure 11. Meanwhile, at the gas outlet section, the FRP slope of the purge curve is initially lower than that of the gas inlet section. However, it remains constant before slowly increases during purging with gas. Finally, the local HFR is close to the overall cell HFR. Investigations show that less water is removed at the gas outlet than that at the gas inlet. Continuing purging with gas increases the removal of water until the water content reaches equilibrium. Therefore, most of the liquid water in the MEA is expected to be removed if the purge process is extended for another 30 to 60 s.

**Figure 12.**
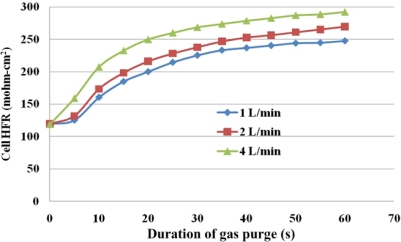
Local HFR variations under various purge gas flow rates—under-the-channel.

**Figure 13.**
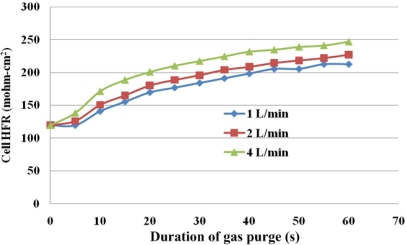
Local HFR variations under various purge gas flow rates—under the rib.

Figures 12 and 13 present local HFR variations for various flow rates of the purging gas. The purging curves tend to differ between the under-the-rib and the under-the-channel areas, indicating that different drying behaviors occur in different areas. Under-the-channel area, a flat SRP stage only takes place at lower purge gas flow rate of 1 L/min. Increasing the flow rate shortens the SRP. When the flow rate is increased to 4 L/min, the HFR is linearly related to the duration of purging for the first 10 s. Thus, the SRP is almost unidentifiable from the purge curve. The sharp slope of FRP takes place which almost takes over the SRP stage. Furthermore, the HFR increases rapidly during the first 30 s of the gas purging procedure and then slowly in the subsequent 30 s.

On the other hand, for under-the-rib area, Figure 13 presents variations of HFR in this area. The significant SRP stage at the low flow rates of 1 and 2 L/min occurs during the first 5 s of gas purging. The HFR rises markedly for the next 5 s and continues to increase until the end of the gas purging process. At a higher flow rate of 4 L/min, the HFR increases to the end of the gas purging, with a larger slope in the SRP than at the lower flow rates, and the FRP is short.

**Figure 14.**
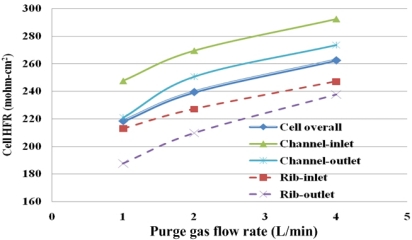
Values of final HFR at different local areas.

Under given purging conditions, the under-the-channel area has a higher HFR than that the under-the-rib area because more water is transported in it via both convection and diffusion. Furthermore, at the same flow rate, the HFR increases slowly in the last 30 s. These results reveal that liquid water in the under-the-channel area is rapidly removed in the first 30 s of gas purging and equilibrium between water content and with the RH of the purging gas may be reached. Meanwhile, the slopes in the SRP and FRP in the under-the-rib area are lower than in the under-the-channel area, and the HFR increases throughout the purging. Accordingly, liquid water that is stored in the under-the-rib area is slowly removed until the end of the purging, and can only be removed by diffusive and evaporative transport. Figure 14 plots the final HFR distributions in areas. The dotted and solid lines are the HFR variations at the under-the-rib area and at the under-the-channel area, respectively. Generally, the final HFR increases with the flow rate. The variation in HFR among various areas declines as the flow rate increases. Thus, a uniform water distribution is obtained in the MEA takes when the flow rate is high enough.

Furthermore, a higher final HFR is measured at the under-the-channel area due to liquid water removal is dominated by both diffusive and convective flux. Increasing the gas flow rate reduces the membrane drying time and enables equilibrium between the water content and the purging gas to be reached. On the other hand, the lowest final HFR value takes place at the under-the-rib area due to the in-plane drying follows the through-plane drying and the liquid water stored at the under-the-rib area may be removed only by diffusion and evaporation transport. Water removal at the under-the-rib area needs longer purge duration. Thus, the purge curve shows a significant SRP in the under-the-rib area and a constant increase in HFR with purge duration after the SRP.

In the along-the-channel direction, regardless of the under-the-channel and the rib areas, a lower HFR value is measured near the gas outlet section, especially at flow rate of 1 L/min. These results may demonstrate that a higher purging gas flow rate cause the gas to reside in the channel for less time. Thus, water content in the purge gas remains low due to less water vapor diffuses into the channel from the MEA and the purge gas RH is stable from the gas inlet section to the outlet section which may further increases the in-plane drying. A lower purge gas flow rate causes the gas to reside for longer in the channel, allowing more vapor to diffuse from the MEA to the flow channel. Water content in the purge gas is increased in the along-the-channel direction which reduces liquid water removal rate. Thus, a significantly lower HFR is measured near the gas outlet, as shown in Figure 14.

### 4.3. Relaxation of HFR

After the gas purge process is stopped, HFR decreases with time. This behavior is called HFR relaxation and was described by Wang [4]. It occurs mainly because of rehydration of the membrane by the residual liquid water after gas purging. However, a fundamental understanding of this mechanism will only be gained from further study.

**Figure 15.**
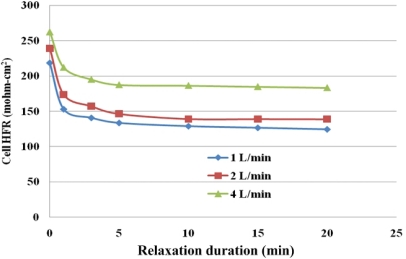
HFR variations after gas purging.

Figure 15 plots HFR relaxation under various flow rates of purging gas. The HFR falls rapidly for the first 5 min and slowly thereafter. Ge and Wang [4] posited possible causes of this phenomenon, such as (1) rehydration of the membrane and ionomers by residual liquid water in the pores in the catalyst layer, the micro porous layer and the GDL after purging; (2) reorganization of the polymer-water structure, particularly in membranes with a low water content; (3) an interfacial non-equilibrium of water between the ionomeric and gas phases, and (4) diffusion of water within the membrane.

**Figure 16.**
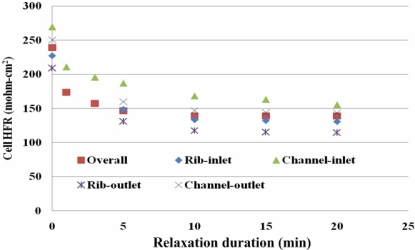
Local HFR relaxation following purging with gas at flow rate of 2 L/min.

HFR relaxation can be reduced by increasing the purge gas flow rate, as displayed in Figure 15. A higher flow rate results in a higher liquid water removal rate and therefore a higher overall cell HFR and local HFR. Thus, the values of HFR decrease slowly due to the residual liquid water in the MEA. Figure 16 plots local HFR relaxation following gas purging at a flow rate of 2 L/min. It clearly indicates the HFR relaxation takes place in the whole MEA in both of the along-the-channel and the across-the-channel directions. The higher HFR after relaxation is measured at the under-the-channel area, and the lower HFR value takes place at the under-the-rib area. Furthermore, at near the gas inlet section, the HFR is higher than those at near the gas outlet section. After gas purge, it is believed that non-equilibrium water content exists especially stored at the under-the-rib area and at near the gas outlet section. The residual water may rehydrate the membrane through the internal diffusion transport. Thus, a more uniform local HFR distribution is measured after the relaxation duration of 20 min.

## 5. Conclusions

This work develops a novel micro sensor device and a consistent and repeatable method for diagnosing *in situ* local water content under various gas purging conditions. The results reveal that the device and method provide accurate sub-millimeter-scale measurement of local HFR variations, supporting a comprehensive fundamental understanding of local area transport phenomena. The most important contributions are as follows.

1. Detailed, local area water content distribution and gas purge phenomena are discovered by dedicated micro sensors. These dedicated micro sensors can be imbeded into a fuel cell and provide more accurate and local area cell performance.
2. Water removal in the MEA can be categorized as through-plane or in-plane drying as shown in purge curves. The purging curve plots HFR *versus* purge duration. A purge curve is composed of three stages, which correspond to particular liquid water removal characteristics in the MEA. They are the slow rise period, the fast rise period, and the membrane equilibrium period. These three stages represent removal of liquid water from the GDL and the catalyst layer, removal of water from the membrane, and equilibrium between water content in the membrane and the purging gas, respectively.
3. Water removal could be identified as convective and diffusive fluxes of water vapor. The flow rate of the purging rate affects both of these fluxes. However, the flow rate dominates the convective flux.
4. Water removal at the under-the-channel area is faster than that at the under-the-rib area, as shown in the purge curves of Figures 12 and 13. In the latter area, liquid water that is stored in the membrane is removed almost immediately at the beginning of purging and then equilibrium of water content may be reached. At the under-the-rib area, a significant in-plane drying takes place and then water removal in the membrane is followed. The membrane equilibrium stage is significantly lower than that at the under-the-channel area. Hence, the purge curve reveals only two stages, SRP and FRP.
5. The HFR relaxation shows the decreasing HFR value at the under-the-rib area is faster than at the under-the-channel area resulting in a lower HFR after gas purge. This indicates non-equilibrium water content exists especially at areas of under the rib and near gas outlet section. The residual water may rehydrate the membrane through internal diffusion transport. Accordingly, the local HFR distribution is more uniform after 20 min.

## Figures and Tables

**Table 1.** Experimental purging with gas.

	**Duration (min)**	**Current density (mA/cm^2^)**	**Gas RH (%)**
Cell initial hydration	60	500	100
Equilibrium purge	120	N/A	75
Liquid water generation	10	500	100
Gas purge	1	N/A	40
Post purge	20	N/A	N/A

**Table 2.** Gas purging conditions.

**Condition**	**Parameters**
Cell temperature (°C)	60
Gas dew point (°C)	41 (40% RH)
Gas flow rate (L/min)	1, 2, 4

**Table 3.** Rate of removal of water at various flow rates of purging gas.

**Gas flow rate (L/min)**	**HFR (mΩ cm^2^)**	**Convective flux (×10^6^ mol/cm^2^ s)**	**Diffusive flux (×10^5^ mol/cm^2^ s)**
1	207	11.32	5.62
2	243	22.63	5.62
4	262	45.27	5.62
